# Two new Marquesan species of the southeastern Polynesian genus *Oparanthus* (Asteraceae, Coreopsidinae)

**DOI:** 10.3897/phytokeys.4.1603

**Published:** 2011-07-12

**Authors:** Warren L. Wagner, David H. Lorence

**Affiliations:** 1 Department of Botany, MRC-166, National Museum of Natural History, Smithsonian Institution, P.O. Box 37012, Washington, DC 20013-7012; 2 National Tropical Botanical Garden, 3530 Papalina Road, Kalaheo, HI 96741 USA

**Keywords:** Asteraceae, conservation, French Polynesia, Marquesas Islands, *Oparanthus*

## Abstract

Two new species of the recently revised genus *Oparanthus* (Asteraceae, Subtribe Coreopsidinae) were discovered during the National Tropical Botanical Garden/Smithsonian Institution 1997 expedition to the Marquesas Islands. *Oparanthus woodii* W. L. Wagner & Lorence, **sp. nov.** is known from a single population on the island of Nuku Hiva, and *Oparanthus tiva* W. L. Wagner & Lorence, **sp. nov.** is known only from Tahuata. Small domatia with a tuft of hairs occur in *Oparanthus tiva* (and the previously known *Oparanthus hivoanus*), and similar but naked domatia are found occasionally in *Oparanthus woodii*. Domatia are of exceedingly rare occurrence in Asteraceae. Both new species are extremely rare and are considered critically endangered (CR) as are the other four species of the genus.

## Introduction

Ongoing investigation of the vascular flora of the Marquesas Islands has been facilitated through a collaboration between National Tropical Botanical Garden (NTBG), Smithsonian Institution (SI), and the Délégation à la Recherche Papeete, Tahiti, French Polynesia. One of the expeditions in 1997 resulted in the discovery of two additional species of the genus *Oparanthus* Sherff, of which there are two previously known Marquesan species and two from Rapa Island (Shannon and Wagner 1997). They are described and illustrated and their conservation status is assessed in this paper.

Since the most recent revision of the genus (Shannon and Wagner 1997) new studies of the phylogeny and biogeography of *Oparanthus* have been completed. *Fitchia* Hook. f., *Oparanthus*, and *Petrobium* R. Br.have been considered to be insular derivatives from the genus *Bidens* L. (Carlquist 1974; Shannon and Wagner 1997). These relationships were not supported by ITS study of the Coreopsideae by Kimball and Crawford (2004). According to their phylogenetic results *Fitchia* and *Oparanthus* are sister genera and share a common ancestor with two Caribbean genera, *Narvalina* Cass. and *Selleophytum* Urb. Another study was initiated (H. Dempewolf, T. Motley, D. Lorence and W. Wagner unpubl.) to further examine the biogeographic patterns in this lineage of two genera with no affinities to other Pacific Asteraceae but which represent an interesting biogeographic disjunction with two genera of the Caribbean. This unpublished phylogenetic analysis of *Oparanthus* and *Fitchia* utilized nuclear ribosomal gene regions (ITS, ETS, and *5S*-NTS). The results of the analyses of four species of *Fitchia* (of eight) and five species of *Oparanthus* (of six) indicate a strongly supported relationship between the two genera and, at least among the extant species, it appears that a *Fitchia/Oparanthus* ancestor first colonized Rapa. This makes sense because Rapa is at the young end of a series of hot-spot traces in southern Polynesia (McNutt et al. 1997). Rapa is about 4.1–4.8 Ma, but the volcanic chains in this region date back to 34-35 Ma (McNutt et al. 1997; Bonneville et al 2002) allowing for the possibility of colonization much earlier than the age of any islands that either genus occurs on presently (i.e., the Societies, Marquesas, Rapa, and Rarotonga).

To date only 47 total collections of *Oparanthus* have been made of the four Marquesan species, which gives an indication of how uncommon they are. Collections of two of the species, *Oparanthus hivoanus* and *Oparanthus teikiteetinii*, consititute the bulk (36). Both of these species occur as scattered individuals in appropriate habitat or are occasionally relatively common in localized areas. The two new species described here are known from only a few collections. When evaluated using the IUCN criteria for endangerment (IUCN 2001) all four of the Marquesan species of *Oparanthus* fall into the Critically Endangered (CR) category, which designates species facing the highest risk of extinction in the wild. Marquesan species of *Oparanthus* meet the IUCN criteria by having known ranges less than 100 km2, an area of occupancy of less than 10 km2, continuing decline in the quality of habitat.

## Methodology

All measurements given herein are taken from dried herbarium specimens, although certain features such as shapes were supplemented with information from alcohol-preserved flowers, field notes, and color slides or digital photos. Measurements are presented in the descriptions as follows: length × width, followed by units of measurement (mm or cm). Specimens from the following herbaria were studied: BISH, K, MO, NY, P, PAP, PTBG, and US.

The new species described here fit well in the sectional classification developed prior to their discovery (Shannon and Wagner 1997). Plants of *Oparanthus* sect. *Albiflori*, which includes all four Marquesan species, are characterized by leaves relatively thin to slightly thickened and subcoriaceous, marginal veins obscure or inconspicuous, the involucral bracts in 2-3 well defined series, corollas white, and achene wings and awns glabrous.

### Key to species of *Oparanthus* in the Marquesas Islands

**Table:** 

1a	Plants hirsute, especially on inflorescences and young shoots and leaves; domatia never present in abaxial primary leaf axils	*Oparanthus teikiteetinii*
1b	Plants glabrous or nearly so; domatia nearly always present in abaxial primary leaf axils	2
2a	Heads solitary; ray floret corolla tube and throat 7.5–8 mm long; leaf bases connate around stem; domatia present or absent in abaxial leaf axils, without associated hairs	*Oparanthus woodii*
2b	Heads in clusters of 3; ray floret corolla tube and throat 3–4 mm long; leaf bases not connate around stem; domatia present in abaxial leaf axils, covered with hairs	3
3a	Trees 3–7 m tall; leaves thinly coriaceous, 10–19.3 × 6–15.8 cm; heads 9–14 mm in diameter, 10–20 mm high; receptacular bracts of ray florets 10–11 mm long, those of disk florets 12–13 mm long; sterile disk achenes 12–13	*Oparanthus tiva*
3b	Shrubs; leaves subcoriaceous, 2.3-8 × 1.3-4.5 cm, on young shoots up to 13 cm long and 11 cm wide; heads 7–12 mm in diameter, 9–13 mm high; receptacular bracts of ray florets 7.5–8 mm long, those of disk florets 9–11 mm long; sterile disk achenes ca. 20	*Oparanthus hivoanus*

#### Oparanthus tiva

W. L. Wagner & Lorence sp. nov.

urn:lsid:ipni.org:names:77112740-1

http://species-id.net/wiki/Oparanthus_tiva

Fig. 1 3C 

##### Latin.

Ab O. hivoano foliis tenue coriaceis, 10-19.3 × 6-15.8 cm, testibus 9-14 mm diametro, 10-20 mm altis, paleis flosculorum radiorum 10-11 mm longis, paleis flosculorum discorum 12-13 mm longis, 12-13 achenis sterili disci differt.

##### Type.

**Marquesas Islands:** Tahuata, ridge E of trail ridge up to Amatea from Kuaee, E facing slope, 2560 ft (780 m), 18 July 1997, S. P. Perlman, K. R. Wood, and J. P. Luce 16008 (Holotype: PTBG-025572!; isotypes: BISH!, P!, PAP!, US!).

##### Description.

*Trees* 3–7 m tall, subglabrous, functionally monoecious, the main trunk to 30 cm in diameter, often with multiple prop roots, bark brown, wood cream colored, the young stems with short internodes. *Leaves* thinly coriaceous, the blade ovate to very broadly ovate, 10–19.3 × 6–15.8 cm, the young ones often viscous and with a turpentine-like scent, secondary veins 6–18 mm apart, abaxial axils with domatia with small tufts of hairs, margins weakly dentate to subentire, apex bluntly acuminate to obtuse, base obtuse to occasionally truncate, often oblique; petioles 3–9 cm long. *Inflorescences* terminal, heads in clusters of 3, 9–14 mm in diameter, 10–20 mm high; peduncles 15–76 mm long; involucre campanulate, the bracts 7–9, in 2 series, 7–11 mm long, the external ones wider and thicker; receptacular bracts of ray florets 10–11 mm long, those of disk florets 12–13 mm long; *ray florets* 8–10 in 1–3 series, white, corolla tube and throat 3–4 mm long, limb 3–3.8 mm long, shallowly toothed; *disk florets* 18–20, white, corolla tube and throat 3.5–4 mm long, the lobes 3 mm long. *Ray achenes* 8–9 mm long, narrowly elliptic to lanceolate, winged on both margins, the wings up to 1 mm wide, the wing margin smooth, extending beyond the achene apex; the disk achenes sterile, linear, 12–13 mm long.

##### Etymology.

We are pleased to name this new species in recognition of its first collector, Steven P. Perlman, known in the Marquesas by his nickname “Tiva”, in recognition of his contributions to our knowledge of the flora of the Pacific region.

##### Distribution.

Endemic to the Marquesas Islands and restricted to the vicinity of the type locality on Tahuata.

##### Ecology.

Scattered to locally common in the low wet forest, dominated by species of *Alsophila*, *Crossostylis*, *Freycinetia*, *Hibiscus*, *Metrosideros*, *Reynoldsia*, and *Weinmannia*, with an understory rich in plants such as *Cyrtandra*, *Dicranopteris*, *Gahnia*, *Macropiper*, *Marattia*, *Morinda*, and *Psychotria*, from 790 to 900 m elevation, usually on windswept slopes and gulches of summit areas. Known to flower and fruit in July.

##### Conservation status.

Following the criteria and categories of IUCN (2001) it is assigned a preliminary status of **Critically Endangered** (CR): B2a, B2b (i-iii); D: B2: total area of occupancy less than 10 km2 (ca. 5 km2). B2a, a single population known; b (i–iii), habitat continuing decline inferred; D, population estimated to number fewer than 250 individuals. The suitable habitat for *Oparanthus tiva* on Tahuata (*c.* 61 km2) is indicated as an endangered environment, threatened feral animals and invasive plants, reducing the extent of the forest. The known habitat is not pristine and non-native plants such as *Ageratum conyzoides* L.*, Elephantopus mollis* Kunth*, Melinis repens* (Willd.) Zizka, *Paspalum conjugatum* P. J. Bergius*, Paspalum paniculatum* L.*, Psidium guajava* L., *Spathoglottis plicata* Blume, and *Zingiber zerumbet* (L.) Sm. were observed in the area.

##### Specimen examined.

**Marquesas Islands:** Tahuata: ridge from Amatea to Moteve passing Meikaea, view down is on village of Hanatetena, on E facing slope, Perlman et al. 15977 (BISH, P, PAP, PTBG, US); off trail from Amatea to Moteve, above Haaoipu Bay, to NE of Hanatetena, top of ridge crest, W facing slope, Perlman et al. 15998 (PTBG); Haaoiputeomo summit region, upper drainage to N of satellite dish, [09 56’S, 139 04’W], Wood 6523 (BISH, MO, NY, P, PAP, PTBG [2], US); Amatea region, locations around Haaoiputeomo satelite dish (parabowl), Wood 10263(PAP, PTBG, US), Wood 10265(PAP, PTBG, US).

##### Discussion.

*Oparanthus tiva* has domatia (with an associated tuft of hairs) on the abaxial surface of the leaves. Domatia are essentially absent in the family, so domatia in *Oparanthus tiva* should be investigated further to understand the ecological significance. When domatia were discovered in *Oparanthus tiva*, we reexamined the other species of the genus and those of the closely related genus *Fitchia*. We found them only in the closest relative, *Oparanthus hivoanus* and in the Nuku Hiva species, *Oparanthus woodii*, but in these without the associated tuft of hairs covering the pit. *Oparanthus tiva* is a relatively rare species with an estimated 100 individuals known. It is distinguished from *Oparanthus hivoanus* by the tree habit, leaves thinly coriaceous, 10–19.3 × 6–15.8 cm, larger heads 9–14 mm in diameter, 10–20 mm high, receptacular bracts of ray florets 10–11 mm long, those of disk florets 12–13 mm long, and the sterile disk achenes 12–13.

**Figure 1.**
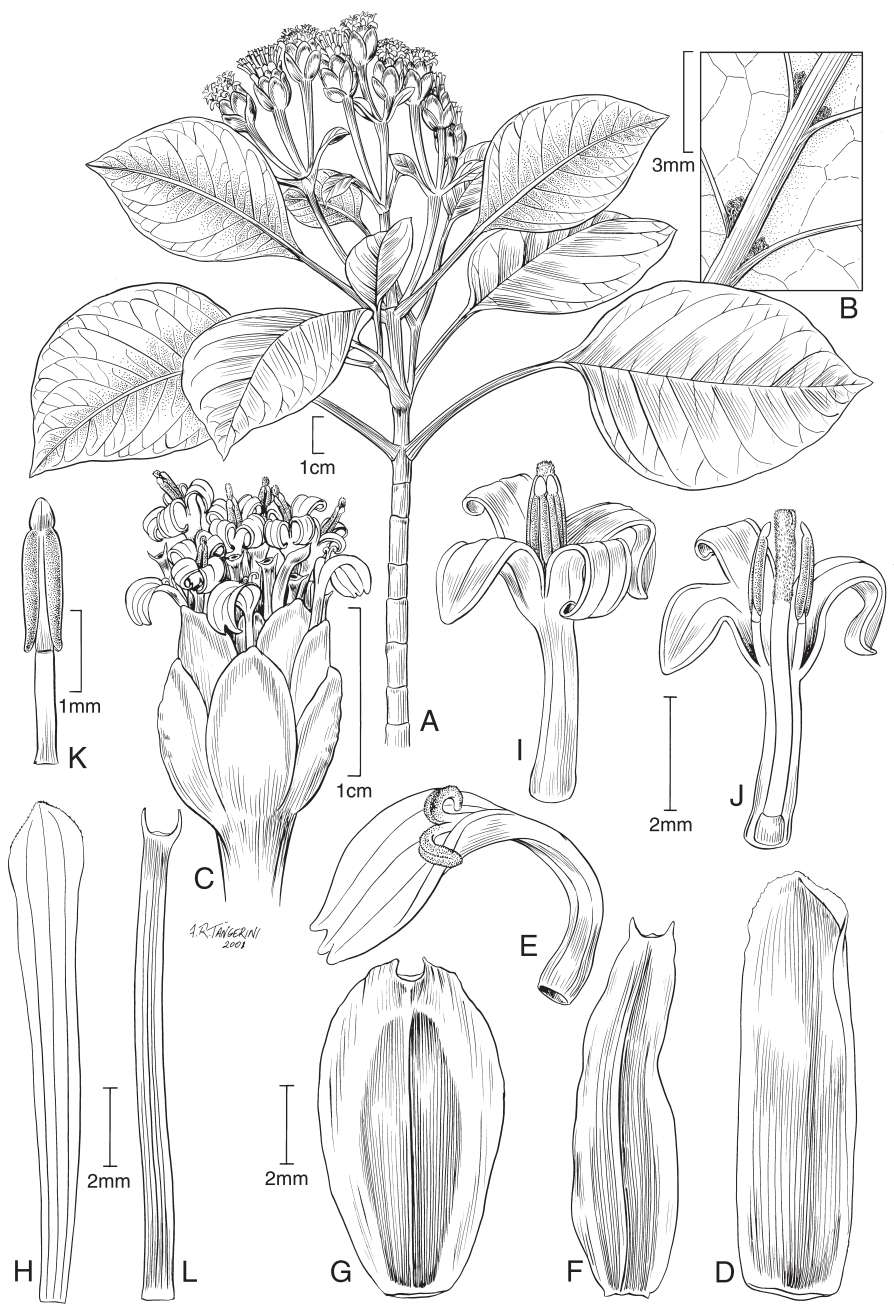
*Oparanthus tiva* W. L. Wagner & Lorence. **A–C** drawn from Perlman et. al 15997, US **A** flowering branch **B** abaxial leaf surface showing domatia with associated tuft of hairs in axils of primary veins **C** head; **D–L** drawn from spirit-preserved material of type collection, Perlman et. al. 16008, PTBG; **D** receptacular bract of ray floret **E** ray corolla and functional style **F–G** ray achene **H** receptacular bract of disk floret **I** disk corolla with non-functional style and fertile stamens **J** longitudinal view of disk corolla with non-functional style and fertile stamens **K** fertile stamen from disk floret **L** sterile disk achene.

#### Oparanthus woodii

W. L. Wagner & Lorence sp. nov.

urn:lsid:ipni.org:names:77112741-1

http://species-id.net/wiki/Oparanthus_woodii

Fig. 2 3D 

##### Latin.

Ab O. teikiteetinii fere glabra arbore, domatiis apilosis in foliis abaxialibus in axilibus nervis secundariis presentibus, foliis basi connatis, flosculis radiorum tubis corollarum 7.5-8 mm differt.

##### Type.

**Marquesas Islands**: Nuku Hiva: Ooumu region, top of Tapueahu Valley off new Hwy, [08 51’53S, 140 10’63W], 1067–1128 m, 23 June 1997, K. R. Wood 6375 (Holotype: PTBG-025565!; isotypes: BISH!, P!, PAP!, US!).

##### Description.

*Trees* 2–5 m tall, glabrous, functionally monoecious, moderately to diffusely branched, the trunk often with multiple prop roots, bark brown, wood cream-colored. *Leaves* thinly coriaceous, the blade broadly elliptic to broadly elliptic-obovate, 11–23.1 × 3.5–12.7 cm, secondary veins 4–12 mm apart, conspicuously arching upward from midrib, then spreading to margin, abaxial axils often with domatia, these without associated hairs, margins entire, apex rounded to truncate, base cuneate, often oblique; petioles 3.5–6 cm long, the base conspicuously connate around stem with paired leaf petiole. *Inflorescences* terminal, heads solitary, 12–16 mm in diameter, 22–35 mm high, peduncles 3–15 mm long, stout; involucre campanulate; involucral bracts 8, in 2 series, the external ones 8–13 mm long, connate at the base, thick and broadly triangular, becoming lignified in fruit, the internal ones usually longer (up to 15 mm) and narrower, triangular to elliptic; receptacular bracts of the ray florets 13–14 mm long, those of the disk florets 12–13 mm long; *ray florets* ca. 18–22, in 2 series, corolla tube and throat 7.5–8 mm long, limb 4.2–4.5 mm long, 2–3-lobed, the lobes usually divided to near corolla throat, occasionally only shallowly so; *disk florets* ca. 40–50 or perhaps more, corolla tube and throat 6.3–6.6 mm long, the lobes 4.4–5.4 mm long. *Ray achenes* elliptic, 5–6 mm long, distinctly winged, the wings ca.1 mm wide, extending slightly beyond the achene apex; disk achenes sterile, linear, 10–11 mm long, with 2 awns.

##### Etymology.

This new species is named for Kenneth R. Wood, who first collected it and who has contributed greatly to our knowledge of the flora of the Marquesas and Hawaii through his collections and field observations.

##### Distribution.

Endemic to Nuku Hiva, Marquesas Islands, and apparently restricted to the Ooumu region, in gulches near the top of Tapueahu Valley, from 1060 to 1130 m elevation.

##### Ecology.

Occurring in montane mesic to wet forest, ravines and steep slopes, with *Metrosideros* and *Weinmannia* dominant and a diverse understory of *Asplenium*, *Blechmum*, *Cyrtandra*, *Hypolepis*, *Ilex*, and *Melicope*, with stands of *Freycinetia* nearby. Known to flower in June, but probably for some months after that.

##### Conservation status.

Based on the IUCN criteria and categories this species is assigned a preliminary Red List status of **Critically** **Endangered** (CR) B2a, B2b (i-iii); D: B2: total area of occupancy less than 10 km2 (ca. 5 km2). B2a, a single population known; b (i–iii), habitat continuing decline inferred; D, population estimated to number fewer than 250 individuals. The suitable habitat for *Oparanthus woodii* on Nuku Hiva (*c.* 340 km2) is indicated as an endangered environment, threatened by human activity (deforestation and fire), feral animals, and invasive plants, thus reducing the extent of the forest.

##### Discussion.

*Oparanthus woodii* appears to be closely related to *Oparanthus teikiteetinii*, which grows at lower elevations, but approaches the known range of *Oparanthus woodii* within a few hundred meters. While *Oparanthus teikiteetinii* is generally distinctive in the genus for its large size, attaining heights of up to 12 m, and for its large, often solitary capitula, *Oparanthus woodii* is a smaller tree up to 5 m tall and has large solitary heads on much shorter and stouter peduncles up to 15 mm long. The corollas are similar in these two species, but the ray corollas of *Oparanthus woodii* are also distinctive in that they are deeply divided to near the corolla throat. Likewise the leaves of *Oparanthus woodii* are distinctive in that they are conspicuously connate at the petiole bases, have secondary veins that arch upwards, and havenaked domatia in the abaxial vein axils. These are not always present and they lack the tufts of hairs in the always present domatia of *Oparanthus hivoanus* and *Oparanthus tiva*.

##### Specimens examined.

**Marquesas Islands:** MARQUESAS ISLANDS: Nuku Hiva, Ooumu region, top of Tapueahu Valley off new Hwy, [08 51’53S, 140 10’63W], Wood et. al. 6338 (P, PTBG, US); Wood 6376 (PTBG, US); Wood 6377 (BISH, K, P, PAP, PTBG, US).

**Figure 2.**
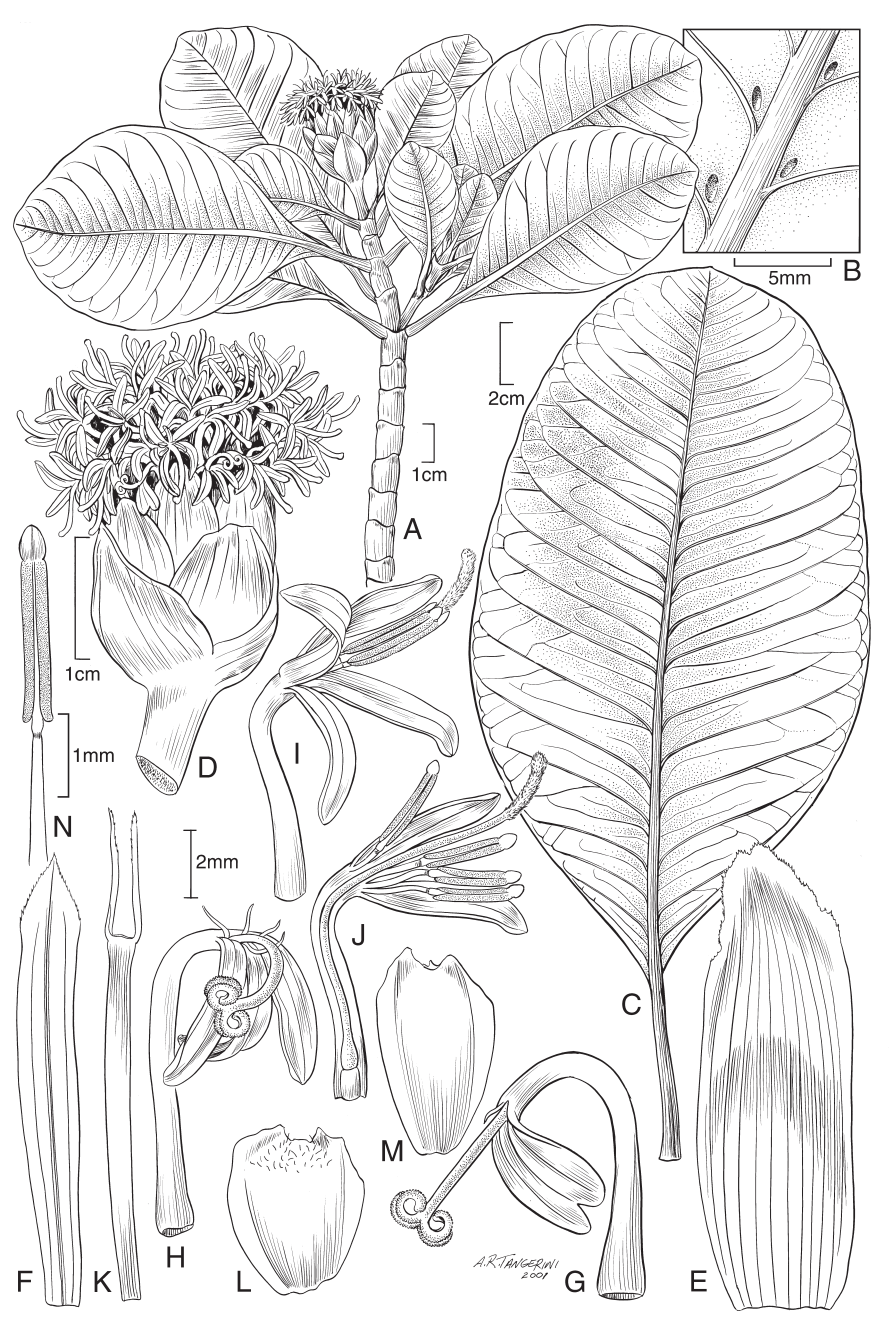
*Oparanthus woodii* W. L. Wagner & Lorence. **A–N** drawn from holotype Wood 6375, PTBG, except **B** from US isotype. **A** flowering branch **B** abaxial leaf surface showing naked domatia in axils of primary veins **C** lower leaf **D** head **E** receptacular bract of ray floret **F** receptacular bract of disk floret **G–H** ray corolla and functional style **I** disk corolla with non-functional style and fertile stamens **J** longitudinal view of disk corolla with non-functional style and fertile stamens **K** sterile disk achene **L–M** ray achenes **N** fertile stamen from disk floret.

**Figure 3.**
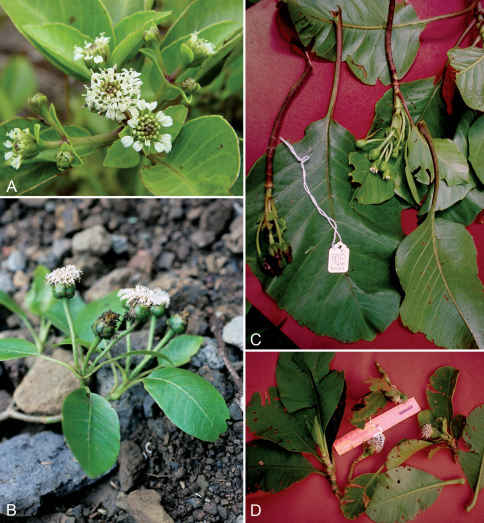
Field images of *Oparanthus*. **A** *Oparanthus hivaoanus*, (Hiva Oa, Price et al 201, photo D. Lorence) **B** *Oparanthus teikiteetinii* (Nuku Hiva, Lorence 6078, photo D. Lorence) **C** *Oparanthus tiva* (Tahuata, Wood 6523, photo K. Wood) **D** *Oparanthus woodii* (Nuku Hiva, Wood 6338, photo K. Wood).

## Supplementary Material

XML Treatment for Oparanthus tiva

XML Treatment for Oparanthus woodii
